# Atlanta, Georgia

**Published:** 1890-08

**Authors:** B. H. Catching

**Affiliations:** Atlanta, Ga.


### CORRESPONDENCE.

ATLANTA, GA., JULY 19, 1890.

*Dear Dr. Taft :—*

Wish you had been at the meeting of the Georgia Dental Society. We met in the mountain city of Gainesville which overlooks the beautiful Blue Ridge mountains. Some thought we would not have a good attendance on account of the meeting being held the week before the Southern, which has just closed a splendid session in this city; but they were mistaken. The Georgia boys turned out well. The attendance was good, the papers not many, but good, the clinics fine. A State meeting never had finer clinics. Let me say before I forget it, that never in the history of any dental society national, State, or local, was the gold used in the clinics obtained from the soil of the place of meeting. About Gainesville are several gold mines, from some of them a part of the gold used was donated, while a part of it was obtained from washings of the very streets of the town. The gold was sent to Mr——————, of New York, who cheerfully refined it, and made it into plate for bridge and crown, and foil. Let this go down on record as the first and only instance of the kind in history. Among the notables present was the beloved father Atkinson of New York—the same genial, whole-souled gentleman of fifty years ago. Father Atkinson contoured a large filling with this gold. A bridge of several teeth was made for one of the leading citizens of the place. Verily are we not rich in resources in this lovely Southland of ours. What we ask is for our northern brethren to come and make our acquaintance. Some of them if they come, will cease to abuse us, and may be love us. Our latchstrings hang out; come and pull them brethren, our doors will fly wide open to you. We had also with us Dr. Gilson, of Boston, a genial, good fellow. He says the scales have fallen from his eyes. We love him and think he reciprocates. One thing is sure his stomach will yearn for the Georgia water melon, and it may be his dreams will be disturbed by visions of a beautiful brunette.

Between the fair damsel and the Georgia water melon we have assurance of Gilson's affection.

The Atkinsonian lecture on microbes was enjoyed by both dentists and laymen. What wonderful mental and physical powers this patriarch possesses. He seems to be as young as when I first saw him, twenty-two years ago—both angels and spirits hover around him.

After the meeting the boys, with as many ladies, excurted to Tallula Falls. Falls not so grand as Niagara, but more beautiful, with mountain peaks and gorges ever presenting to the eye kaleidoscopic views of the most fascinating character.

I should not forget the banquet graced by the presence of Gainesville's beauty and chivalry, where young hearts swelled while the old renewed their youth. The orator was there, from the judicial bench to the young limb of the law down to the Ciceroes in dentistry.

Come next time Mr. Editor, and bring others. We are to meet on one of the islands of the Atlantic where you can breathe the spice-laden air from the Antilles and bathe in the ever restless ocean. Dr. R. B. Adair will wield the gavel.

Yours, B. H. CATCHING.

